# Supplementary material for: FERN – a Java framework for stochastic simulation and evaluation of reaction networks
Source: BMC Bioinformatics. 2008 Aug 29;9:356. doi: 10.1186/1471-2105-9-356 (PMC2553347; doi:10.1186/1471-2105-9-356)
Supplement: Additional file 1 — FERN distribution, Version 1.3. This archive contains the FERN source code and binaries as well as documentation and example models in FernML and SBML. [file 1471-2105-9-356-S1.zip › fern/doc/javadoc/fern/simulation/observer/Observer.html]

Observer


---


|  |  |  |
| --- | --- | --- |
| **Overview** \| **Package** \| **Class** \| **Use** \| **Tree** \| **Deprecated** \| **Index** \| **Help** | |  |
| **PREV CLASS**   **NEXT CLASS** | **FRAMES**    **NO FRAMES**     **All Classes** |  |
| SUMMARY: NESTED \| FIELD \| CONSTR \| METHOD | DETAIL: FIELD \| CONSTR \| METHOD |  |


---


## fern.simulation.observer Class Observer

```
java.lang.Object
  fern.simulation.observer.Observer
```

**Direct Known Subclasses:**: AmountAtMomentObserver, CellGrowthObserver, CytoscapeColorChangeObserver, FireTypeObserver, InstantOutputObserver, IntervalObserver, LeapObserver, RandomNumberGeneratorCallObserver, TriggerObserver

---

``` public abstract class Observer extends Object ```

Implementing classes are used to observe certain aspects of simulations. It is basically
just an interface of methods that get called during a simulation together with some
common methods useful for many types of observers.

Some observers take repeats into account. If you repeat the simulations, they will
yield average data.

Each observer should override the `toString` method which should return
a string representation of its data.

You can register a `PrintWriter` to the `Observer`. If you have, the
return value of `toString` is printed if a simulation has finished.

**Author:**
:   Florian Erhard

---

**Constructor Summary**

`Observer(Simulator sim)`
  
          Creates an observer dedicated to one simulator.


| **Method Summary** | |
| --- | --- |
| `abstract  void` | `activateReaction(int mu, double tau, Simulator.FireType fireType, int times)`             Gets called before a reaction fires. |
| `protected  String[]` | `applyLabelFormat(String[] name)`             Applies the actual label format to the whole array (but does not change the passed array, it return a new one) |
| `abstract  void` | `finished()`             Gets called when a simulation has finished, directly after the termination check. |
| `protected  String` | `getLabel(String label)`             Formats a label with the actual label format: %l is replaced by the passed argument %a is replaced by the simulator's name %n is replaced by the network's name |
| `String` | `getLabelFormat()`             Gets the label format of `getLabel(String)`. |
| `int` | `getNumSimulations()`             Gets the number of finished repeats of the simulation. |
| `PrintWriter` | `getPrintWriter()`             Gets the registered `PrintWriter` |
| `Simulator` | `getSimulator()`             Gets the simulator |
| `double` | `getTheta()`             Gets the moment in time, where the simulator has to invoke `theta(double)`. |
| `protected  String` | `getTitlesCommand(String[] titles)`             Gets a gnuplot command if you want to plot a histogram and have the data labels for each value in an array |
| `void` | `print()`             Gets called after the `finish` call and prints the results of this observer if a `PrintWriter` is registered. |
| `void` | `setLabelFormat(String labelFormat)`             Sets the label format for `getLabel(String)`. |
| `void` | `setPrintWriter(PrintWriter printWriter)`             Registers a `PrintWriter` |
| `protected  void` | `setSimulator(Simulator simulator)`             Sets the simulator. |
| `void` | `setTheta(double theta)`             Sets the moment in time, where the simulator has to invoke `theta(double)`. |
| `abstract  void` | `started()`             Gets called when the simulation has started after the initialization and before the termination condition is checked the first time. |
| `abstract  void` | `step()`             Gets called after each termination check and before `Simulator.performStep(fern.simulation.controller.SimulationController)` is called. |
| `abstract  void` | `theta(double theta)`             Gets called by simulators when a certain moment in time is reached. |

**Methods inherited from class java.lang.Object**

`clone, equals, finalize, getClass, hashCode, notify, notifyAll, toString, wait, wait, wait`

**Constructor Detail**

### Observer

```
public Observer(Simulator sim)
```

:   Creates an observer dedicated to one simulator. The observer is NOT registered at the
    simulator, you have to call `Simulator.addObserver(Observer)` in order to do this.

    **Parameters:**: `sim` - the simulator


**Method Detail**

### started

```
public abstract void started()
```

:   Gets called when the simulation has started after the initialization and before
    the termination condition is checked the first time.

---


### step

```
public abstract void step()
```

:   Gets called after each termination check and before `Simulator.performStep(fern.simulation.controller.SimulationController)`
    is called.

---


### finished

```
public abstract void finished()
```

:   Gets called when a simulation has finished, directly after the termination check.

---


### theta

```
public abstract void theta(double theta)
```

:   Gets called by simulators when a certain moment in time is reached. This moment in
    time has to be registered by `getTheta()`

    :   **Parameters:**: `theta` - moment in time

---


### activateReaction

```
public abstract void activateReaction(int mu,
                                      double tau,
                                      Simulator.FireType fireType,
                                      int times)
```

:   Gets called before a reaction fires.

    :   **Parameters:**: `mu` - the reaction which is supposed to fire: `tau` - the time the reaction fires (at this time `Simulator.getTime()` does not necessarily yield the firing time): `fireType` - the type of the firing: `times` - TODO

---


### print

```
public void print()
```

:   Gets called after the `finish` call and prints the results of this
    observer if a `PrintWriter` is registered.

---


### getTheta

```
public double getTheta()
```

:   Gets the moment in time, where the simulator has to invoke `theta(double)`.

    :   **Returns:**: theta

---


### setTheta

```
public void setTheta(double theta)
```

:   Sets the moment in time, where the simulator has to invoke `theta(double)`.

    :   **Parameters:**: `theta` - the theta

---


### getPrintWriter

```
public PrintWriter getPrintWriter()
```

:   Gets the registered `PrintWriter`

    :   **Returns:**: the `PrintWriter`

---


### setPrintWriter

```
public void setPrintWriter(PrintWriter printWriter)
```

:   Registers a `PrintWriter`

    :   **Parameters:**: `printWriter` - the `PrintWriter`

---


### setLabelFormat

```
public void setLabelFormat(String labelFormat)
```

:   Sets the label format for `getLabel(String)`.

    :   **Parameters:**: `labelFormat` - the label format **See Also:**: `getLabel(String)`

---


### getLabelFormat

```
public String getLabelFormat()
```

:   Gets the label format of `getLabel(String)`.

    :   **Returns:**: labelFormat the label format **See Also:**: `getLabel(String)`

---


### applyLabelFormat

```
protected String[] applyLabelFormat(String[] name)
```

:   Applies the actual label format to the whole array (but does not change the
    passed array, it return a new one)

    :   **Parameters:**: `name` - array of labels **Returns:**: array of formatted labels

---


### getTitlesCommand

```
protected String getTitlesCommand(String[] titles)
```

:   Gets a gnuplot command if you want to plot a histogram and have
    the data labels for each value in an array

    :   **Parameters:**: `titles` - data labels for each histogram value **Returns:**: gnuplot command to label the x axis

---


### getLabel

```
protected String getLabel(String label)
```

:   Formats a label with the actual label format:

    - %l is replaced by the passed argument
    - %a is replaced by the simulator's name
    - %n is replaced by the network's name

    :   **Parameters:**: `label` - label to format **Returns:**: formatted label

---


### getSimulator

```
public Simulator getSimulator()
```

:   Gets the simulator

    :   **Returns:**: simulator

---


### setSimulator

```
protected void setSimulator(Simulator simulator)
```

:   Sets the simulator. For internal use only. If you want to override it, be careful
    with the consistency between the simulator's observer list and the observer's simulator!

    :   **Parameters:**: `simulator` - the simulator

---


### getNumSimulations

```
public int getNumSimulations()
```

:   Gets the number of finished repeats of the simulation. The number is increased
    after the `finished` call, so within `finish` of an extending
    class it is the number of repeats without the actual simulation.

    :   **Returns:**: number of repeats


---


|  |  |  |
| --- | --- | --- |
| **Overview** \| **Package** \| **Class** \| **Use** \| **Tree** \| **Deprecated** \| **Index** \| **Help** | |  |
| **PREV CLASS**   **NEXT CLASS** | **FRAMES**    **NO FRAMES**     **All Classes** |  |
| SUMMARY: NESTED \| FIELD \| CONSTR \| METHOD | DETAIL: FIELD \| CONSTR \| METHOD |  |


---
